# Antibody Assay and Anti-Inflammatory Function Evaluation of Therapeutic Potential of Different Intravenous Immunoglobulins for Alzheimer’s Disease

**DOI:** 10.3390/ijms24065549

**Published:** 2023-03-14

**Authors:** Zhangcheng Fei, Renjun Pei, Bo Pan, Shengliang Ye, Rong Zhang, Li Ma, Zongkui Wang, Changqing Li, Xi Du, Haijun Cao

**Affiliations:** Institute of Blood Transfusion, Chinese Academy of Medical Sciences and Peking Union Medical College, Chengdu 610052, China; zhangchengfei94@163.com (Z.F.);

**Keywords:** intravenous immunoglobulin, Alzheimer’s disease, β-amyloid, tau, inflammation

## Abstract

Alzheimer’s disease (AD) is a common neurodegenerative disease that currently has no known cure. Intravenous immunoglobulin (IVIG), which contains AD-related antibodies and has anti-inflammatory properties, has shown potential as a treatment for AD. However, the efficacy of clinical trials involving AD patients treated with IVIG has been inconsistent. Our previous study found that different IVIGs had significantly varied therapeutic effects on 3xTg-AD mice. In order to investigate the relationship between the composition and function of IVIG and its efficacy in treating AD, we selected three IVIGs that showed notable differences in therapeutic effects. Then, the concentrations of specific antibodies against β-amyloid (Aβ)_42_, tau, and hyperphosphorylated tau (p-tau) in three IVIGs, as well as their effects on systemic inflammation induced by lipopolysaccharide (LPS) in Balb/c mice, were analyzed and compared in this study. The results indicated that these IVIGs differed greatly in anti-Aβ_42_/tau antibody concentration and anti-p-tau ratio, and improved LPS-stimulated peripheral inflammation, liver and kidney injury, and neuroinflammation in Balb/c mice to varying degrees. Combined with our previous results, the efficacy of IVIG against AD may be positively correlated with its level of AD-related antibodies and anti-inflammatory ability. AD-related antibody analysis and functional evaluation of IVIG should be given sufficient attention before clinical trials, as this may greatly affect the therapeutic effect of AD treatment.

## 1. Introduction

Alzheimer’s disease (AD) is the most common neurodegenerative disease, leading to gradual cognitive impairment and personality abnormalities [1]. The prevalence of AD is increasing year by year as the world population ages [2,3]. It is anticipated that the number of AD patients will approach 130 million by 2050, putting a significant psychological load on patients’ relatives while also resulting in massive social and public expenditure [4,5]. Given the lack of a definitive cure for AD, the development of preventive strategies and therapeutic interventions is of paramount importance.

Intravenous immunoglobulin (IVIG) is a human plasma-derived therapeutic preparation composed of polyclonal IgG (>95%) purified from the pooled plasma of thousands of healthy donors [6]. It has been used clinically for nearly 40 years, and its safety has been well recognized [7]. IVIG not only contains anti-β-amyloid (Aβ) and anti-tau antibodies, but it also possesses anti-inflammatory and immunomodulatory functions [8,9,10]. Therefore, IVIG has been considered as a potential drug for the treatment of AD. Since 2013, at least five randomized controlled studies of IVIG for patients with AD have been conducted around the world [11,12,13,14,15]. However, IVIG has showed inconsistency as a potential treatment for AD in these clinical trials. The underlying reason is unknown, but the differences in IVIG used in these clinical trials have received insufficient attention.

Although the pathogenesis of AD is still unclear, it is considered that the most important pathophysiological characteristics are extracellular Aβ deposition to form neuritic plaques and intracellular hyperphosphorylated tau (p-tau) protein to form neurofibrillary tangles [16]. Growing evidence suggests that inflammation appears to have an essential role in the development of AD [17]. As such, the level of anti-AD-related antibodies and the anti-inflammation function of IVIG may be vital for its treatment in AD. In order to determine whether the difference of IVIG would affect its efficacy in the treatment of AD, we explored the neuroprotective effects of IVIGs from various manufacturers on triple-transgenic (3xTg-AD) mice in our previous study [18]. It has been confirmed that the efficacy of these IVIGs on 3xTg-AD mice is significantly different. In terms of the efficacy of IVIG on 3xTg-AD mice, IVIG-C is greater than IVIG-A, and IVIG-A is greater than IVIG-B (the results will be published soon).

In this work, three IVIGs that showed significantly different therapeutic effects (IVIG-A/B/C) for AD in our previous study were selected to detect and compare the concentrations of specific antibodies to different conformations of soluble Aβ_42_, tau, and p-tau. Moreover, we analyzed the effects of these IVIGs on systemic inflammation stimulated by lipopolysaccharide (LPS) in mice. The aim of this study was to explore whether the AD-related antibody level and anti-inflammatory ability of IVIG were related to its efficacy in the treatment of AD.

## 2. Results

### 2.1. Aβ_42_ Monomer and Soluble Oligomers

**Figure 1:**
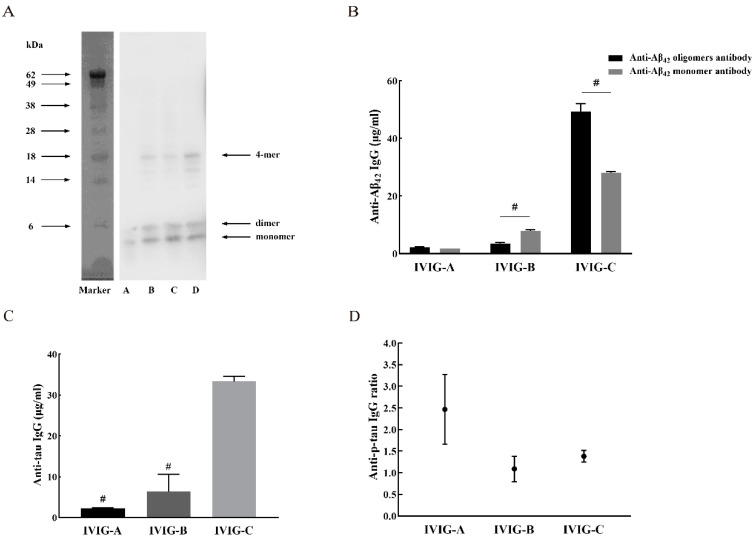
(**A**) WB of Aβ_42_ preparations was generated in various conditions. Lane A: monomeric Aβ (MW 4.5 kDa) generated by resuspending lyophilized Aβ_42_ in TFA and HFIP to disaggregate it, followed by dissolving it in TFA water (pH 3.0) and adding Tris base and HCl to adjust the pH to 8.8. Lane B: Aβ_42_ oligomers were generated by resuspending disaggregated Aβ_42_ in DMSO, followed by bath sonication and aggregation for 3 days. Lanes C and D: Aβ_42_ oligomers with aggregation for 5 days and 6 days, respectively. (**B**) Mean concentrations of antibodies to Aβ_42_ monomer and oligomers in IVIG. Statistical significance using one-way ANOVA is defined as # *p* < 0.001. (**C**) Results for anti-tau antibody concentrations for IVIG products are shown. Statistical significance using one-way ANOVA defined as # *p* < 0.001 vs. IVIG-C group. (**D**) Results for anti-p-tau antibody ratios for IVIG products are shown. Three experiments were performed on each IVIG. Data are expressed as the mean ± SEM.

The prepared Aβ_42_ monomer and soluble oligomers were detected and confirmed by Western blots (WB). A WB of Aβ_42_ monomer and oligomers subjected to gel electrophoresis under reducing and denaturing conditions is shown in Figure 1A. When Aβ_42_ was dissolved in TFA water (pH 3.0) and the pH was then adjusted to 7.0, only one band was generated in SDS-PAGE gels (Figure 1A, lane A). This was assumed to represent monomeric Aβ_42_ (molecular weight = 4.5 kDa). To achieve soluble Aβ_42_ oligomers, disaggregated Aβ_42_ peptide was resuspended in DMSO and diluted in PBS with SDS. After aggregation for 3 days (Figure 1A, lane B), 5 days (Figure 1A, lane C), and 6 days (Figure 1A, lane D), multiple oligomeric bands of Aβ_42_ species were visualized on the SDS-PAGE gel. Aβ_42_ oligomers preparations contained abundant monomer and dimer and some low-order oligomers with a molecular weight of 14~28 kDa, probably representing Aβ_42_ trimer to 4-mer molecules.

### 2.2. Anti-Aβ_42_ Antibody Concentration in IVIG

Oligomers polymerized for 6 days were used for antibody detection given their greater oligomer conformation. The concentrations of anti-Aβ_42_ monomer and oligomer antibodies in IVIG are shown in Figure 1B. The concentration of anti-Aβ_42_ monomer antibodies in the tested IVIG were 1.8 μg/mL (IVIG-A), 7.9 μg/mL (IVIG-B), and 28.0 μg/mL (IVIG-C), and anti-Aβ_42_ oligomer antibodies concentrations were 2.3 μg/mL (IVIG-A), 3.5 μg/mL (IVIG-B), and 49.4 μg/mL (IVIG-C). There were significant differences in anti-Aβ_42_ antibody concentration among the three IVIGs and the antibody concentration in IVIG-C was the highest. The concentrations of Aβ_42_ monomer antibody were significantly lower than the Aβ_42_ oligomer antibody in IVIG-C (*p* < 0.0001), while the opposite was observed for IVIG-B (*p* < 0.001).

### 2.3. Anti-Tau Antibody Concentration and Anti-P-Tau Ratio in IVIG Preparations

Specific anti-tau antibodies were detected in each of the IVIGs. The mean anti-tau levels were 2.3 μg/mL for IVIG-A, 6.4 μg/mL for IVIG-B, and 33.3 μg/mL for IVIG-C (Figure 1C). The anti-tau antibody concentration in IVIG-C was significantly higher than IVIG-A (*p* < 0.0001) and IVIG-B (*p* < 0.0001). The estimated means for anti-p-tau antibody ratios for the IVIG products were 2.5 for IVIG-A, 1.1 for IVIG-B, and 1.4 for IVIG-C (Figure 1D). All three IVIGs contain antibodies against p-tau, but the ratio is different.

### 2.4. Effects of IVIG on LPS-Stimulated White Blood Cell, TNF-α, and IL-6 in the Serum

In this study, we employed Balb/c mice to investigate the impact of IVIG on systemic inflammation. To induce a systemic inflammatory response, we administered LPS to mice, and this group served as the Model group. The inflammatory model of mice treated with IVIG was used as the IVIG (IVIG-A/B/C) treatment group, while the control (Ctrl) group was injected with normal saline only (details in Section 4.4).

**Figure 2:**
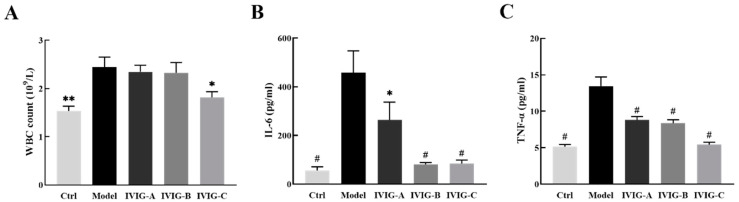
Effect of IVIG on WBC count in mice with LPS (**A**). More WBC counts were noted in the serum of Model group than that of Ctrl group and this increase was markedly attenuated after treatment with IVIG-A but not IVIG-B or IVIG-C. IVIG inhibits the release of (**B**) IL-6 and (**C**) TNF-α in LPS-stimulated Balb/c mice. Ctrl, control group; Model, LPS injection (Model) group; TNF-α, tumor necrosis factor-α; IL-6, interleukin-6; Data are expressed as the mean ± SEM. Statistical significance using one-way ANOVA defined as * *p* < 0.05, ** *p* < 0.01, ^#^ *p* < 0.001 vs. Model group (*n* = 6).

The levels of white blood cells (WBCs), TNF-α, and IL-6 in the serum are shown in Figure 2. In the present study, the WBC count was observed to be markedly increased in the serum of Model group compared with Ctrl group (Figure 2A, *p* < 0.01). Compared with Model group, treatment with IVIG-C reduced the WBC count (Figure 2A, *p* < 0.05), while WBC count in the IVIG-A and IVIG-B groups did not change significantly. As presented in Figure 2B,C, the increase of TNF-α and IL-6 concentrations was confirmed by the administration of LPS (*p* < 0.001). The increase was significantly attenuated by pretreatment with three IVIGs (*p* < 0.05). These results demonstrated that three IVIGs exerted an inhibitory effect on LPS-stimulated inflammation to different degrees. IVIG-C showed better performance in inhibiting the increase of WBC and inflammatory factors in the blood.

### 2.5. Effects of IVIG on LPS-Stimulated Histopathological Score in the Liver and Kidney

**Figure 3:**
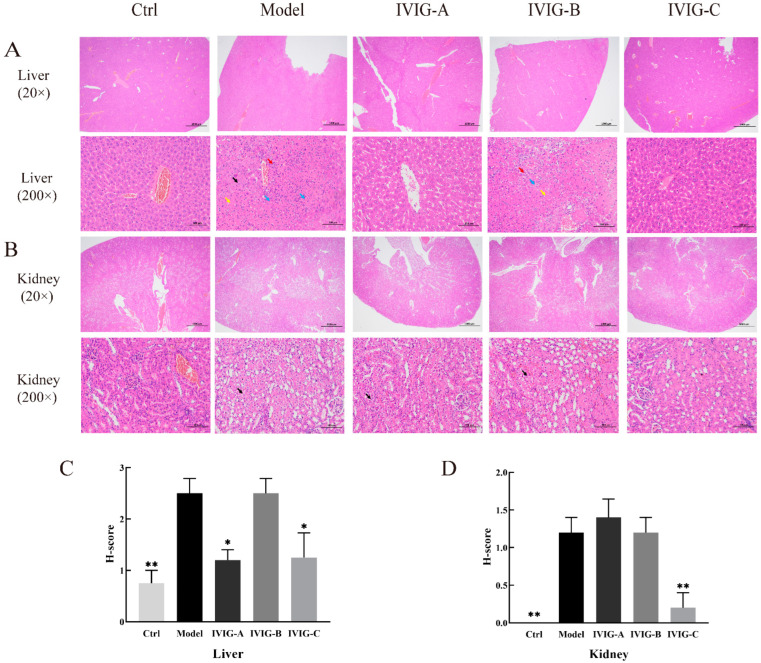
The histopathological changes of the liver (**A**) and kidney (**B**). IVIG exerted different degrees of ameliorating effects on the liver and kidney damage. The black arrow indicates mild watery degeneration of the cell, swelling of the cell, and loose and bright cytoplasm; The yellow arrow indicates focal necrosis and karyolysis of hepatocytes; The red arrow indicates a small amount of connective tissue hyperplasia; The blue arrow indicates punctate lymphocyte and neutrophil infiltration. The H-score was used to grade the severity of the lesion of the liver (**C**) and kidney (**D**). Ctrl, control group; Model, LPS injection (Model) group; Data are expressed as the mean ± SEM. Statistical significance using one-way ANOVA defined as * *p* < 0.05, ** *p* < 0.01 vs. Model group (*n* = 4).

The histopathological properties of LPS-stimulated liver and kidney are shown in Figure 3. Histopathological analysis showed that exposure to LPS caused watery degeneration of hepatocytes, swelling of cells, loose cytoplasm, and light staining widely in the liver tissue (Figure 3A,C). These changes increased the liver histopathological score (H-score) (*p* < 0.01). In contrast, IVIG-A and IVIG-C alleviated these changes and reduced liver H-score (*p* < 0.05; Figure 3A,C). Stimulation of LPS caused mild watery degeneration, swelling of cells, loose cytoplasm, and light staining of a large number of renal tubular epithelial cells in the corticomedullary junction of the mouse kidney tissue (*p* < 0.01; Figure 3B,D). Only just IVIG-C alleviated these changes and reduced liver H-score (*p* < 0.05; Figure 3B,D). These results demonstrated that three IVIGs exerted different degrees of ameliorating effects on LPS-stimulated liver and kidney damage in Balb/c mice. Only IVIG-C simultaneously suppressed LPS-stimulated liver and kidney damage.

### 2.6. Effects of IVIG on LPS-Stimulated Histopathological Score in the Brain and Hippocampus

**Figure 4:**
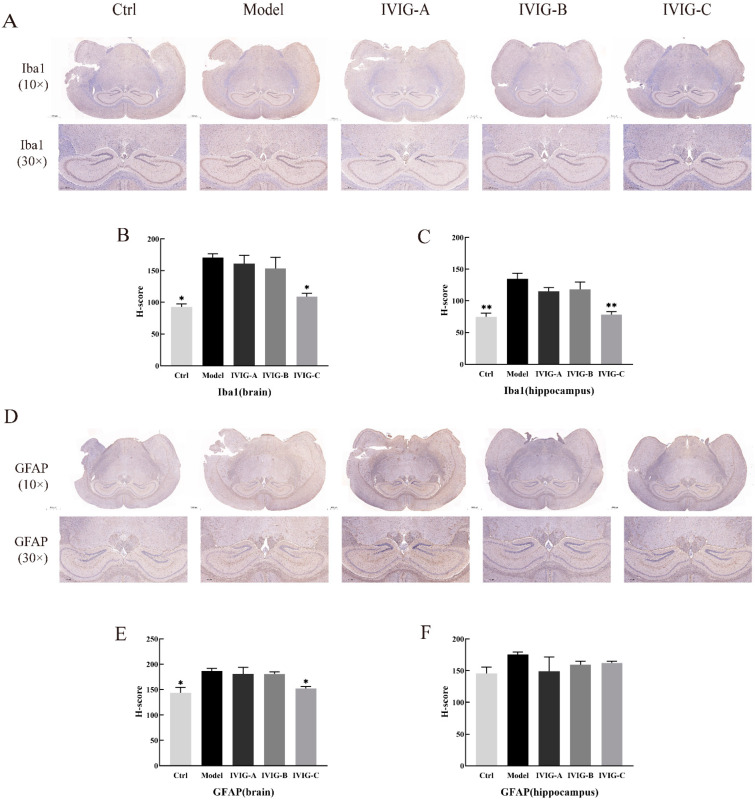
(**A**) Iba1-positive cells in the brain and hippocampus. H-score was used to grade the percentage of IBA-1 positivity in the brain (**B**) and hippocampus (**C**). IVIG-C reversed the increase of IBA-1 positive cells in the brain and hippocampus of mice induced by LPS. (**D**) GFAP-positive cells in the brain and hippocampus. H-score was used to grade the percentage of GFAP positivity in the brain (**E**) and hippocampus (**F**). IVIG-C reversed the increase of IBA-1-positive cells in the brain of mice induced by LPS. Ctrl, control group; Model, LPS injection (Model) group; Data are expressed as the mean ± SEM. Statistical significance using one-way ANOVA defined as * *p* < 0.05, ** *p* < 0.01 vs. Model group (*n* = 3–4).

Intraperitoneal LPS in mice has been shown to induce neuroinflammation, which is characterized by the activation of microglia and astrocytes in the brain [19,20]. Given that the hippocampus is an important organ involved in learning and memory storage in the central nervous system, this study focused on the protective effects of IVIG on the brain and hippocampus. Iba1 and GFAP are specific biomarkers of activated microglia and astrocytes, respectively [21]. The histopathological properties of the LPS-stimulated brain and hippocampus are shown in Figure 4. More Iba1 and GFAP-positive cells were noted in the brain of Model group than that in Ctrl group (*p* < 0.05; Figure 4A,B,D,E) and this increase was markedly attenuated after IVIG-C (*p* < 0.05; Figure 4A,B,D,E) treatment. In the hippocampus, there are more Iba-1-positive cells in Model group than that in Ctrl group (*p* < 0.01; Figure 4A,C,D,F) and the number of Iba-1-positive cells was reduced by IVIG-C (*p* < 0.01; Figure 4A,C,D,F). There was no significant difference in GFAP-positive cells in the hippocampus among all groups (Figure 4D,F). The levels of Iba-1 and GFAP-positive cells were not decreased by IVIG-A or IVIG-B in the brain or hippocampus (Figure 4A–F). These results demonstrated that three IVIGs exerted different degrees of ameliorating effects on LPS-stimulated neuroinflammation in Balb/c mice. Only IVIG-C suppressed LPS-stimulated neuroinflammation.

## 3. Discussion

The existence of AD-related antibodies and anti-inflammatory properties in IVIG makes it a potential treatment for AD [22,23,24]. However, satisfactory results have not been duplicated in several clinical trials. In the phase II clinical trial with IVIG Ocapharma^TM^ [12], no significant difference was found in the cognitive and functional scores between the treatment group and the placebo group. In the phase III trial of AD patients treated with IVIG Gammagard^TM^, there was no significant difference in the measures of cognitive functioning between the IVIG group and the placebo group [25]. In 2017, Grifols conducted a phase II randomized controlled trial involving 52 patients with mild AD [11]. The brain atrophy and cognitive function were significantly improved after 12 months of IVIG treatment. IVIG used in these clinical trials is produced by different manufacturers and the impact of IVIG itself cannot be ignored. Therefore, the inconsistency of results in these clinical trials prompted us to turn our attention to IVIG itself. Previously, we explored the neuroprotective effects of different IVIG in 3xTg-AD mice and found that the efficacy against AD was significantly different [18]. Three IVIG with significant differences in efficacy in 3xTg-AD mice were selected to further analyze their Aβ_42_ and tau/p-tau antibody levels and evaluate their anti-inflammatory function in this study.

Aβ_42_ is believed to be an early and important contributor to AD pathogenesis among the isoforms of Aβ, and it is found as monomers and soluble oligomers [26]. Aβ_42_ is the main pathogenic and aggregation-prone subspecies that triggers a cascade of downstream injuries leading to neuronal dysfunction and degeneration [27]. As a microtubule-associated protein, tau stabilizes the neuronal cytoskeleton, and its hyperphosphorylation may be a primary driver of neurodegeneration in AD [28]. In this study, a significant difference was found in the concentration of anti-Aβ_42_ and anti-tau antibodies among the three IVIGs, and the antibody concentration in IVIG-C was higher than that in IVIG-A and IVIG-B. All three IVIGs contained anti-p-tau antibodies, but the proportion varied greatly.

The emergence and development of systemic inflammatory response is also an early pathological alteration in AD [29]. Neuroinflammation has been proven to be an important promoting factor in the progression of AD, and it is mainly reflected in the activation of microglia and astrocytes in the hippocampus or whole brain [20,30]. LPS injection has been shown to initiate an inflammatory response that leads to an increase in WBCs, and this causes the production of pro-inflammatory cytokines and consequent damage to organs throughout the body [31,32]. The mice in this study showed systemic inflammation-like biochemical and physiological changes after receiving 1 mg/kg LPS. LPS-stimulated Balb/c mice showed not only a significant increase in levels of WBC, IL-6, and TNF-α, but also inflammatory reaction and injury in the liver, kidney, brain, and hippocampus. Regulating the homeostasis of WBC and inhibiting the release of inflammatory cytokines are the key to reducing the deterioration of systemic inflammation [33]. All three IVIGs successfully inhibited the excessive release of IL-6 and TNF-α in the serum, but only IVIG-C improved the increase in the number of WBCs. The degree of organ damage indicates the severity of inflammation [34]. In this study, H-score was chosen to assess the extent of the liver, kidney, brain, and hippocampus injury. There were obvious mild inflammatory changes in the liver, kidney, brain, and hippocampus of mice in the model group, and three IVIGs reversed this pathology to varying degrees. Only IVIG-C not only alleviated the histopathological changes in the liver and kidney, but also ameliorated the overactivation of microglia and astrocytes in the brain and hippocampus. IVIG-A and IVIG-B improved inflammatory lesions in the kidney, but this benefit was not observed in the liver, brain, or hippocampus.

The results suggested that the concentrations of anti-Aβ_42_/tau antibody, anti-p-tau ratio, and anti-inflammatory ability differ greatly among the three IVIGs. These differences likely explain the varying efficacy of IVIG observed in animal experiments. We tracked the production and preparation of these three IVIGs and found these IVIGs were produced by different manufacturers located in north, northwest, and south China. Each manufacturer has its own plasma collecting site, and the donors who come to donate plasma vary in race and geography [8]. Furthermore, there are 56 ethnic groups living in the vast land of China [35,36]. The genetic background, geographical distribution, and diet of donors may contribute to the unique composition of each donor’s immunoglobulin repertoire [37]. In addition, the preparation processes of IVIG by different manufacturers are not identical, which may also affect the composition of IVIG. These reasons may together contribute to the discrepancy in the composition of IVIG produced by different manufacturers, and thus lead to differences in its biological functions.

In the previous animal study, IVIG-C surprisingly showed better potential than IVIG-A and IVIG-B in the treatment of AD. In this study, IVIG-C not only had a higher concentration of anti-Aβ and anti-tau antibodies, but also exhibited a better ability to inhibit systemic and cerebral inflammation. We speculated that the efficacy of IVIG against AD was positively correlated with its level of AD-related antibodies and anti-inflammatory ability. However, it is yet to be determined which factor is more essential. The level of AD-related antibody in IVIG-B was higher than that in IVIG-A, but IVIG-A exhibited slightly better anti-inflammatory ability. This finding may explain why IVIG-A was more effective in inhibiting the release of inflammatory factors in 3xTg-AD mice.

Therefore, antibody analysis and functional evaluation of IVIG are extremely essential before clinical trials for AD treatment, and preclinical evaluation criteria for the potential therapeutic efficacy of IVIG against AD need to be established. In addition, IVIG produced by certain manufacturers (IVIG-C) had high antibody components and anti-inflammatory ability, and performed better in the treatment of AD. This suggested that the different components and functions of IVIG have a great influence on the efficacy of AD. The development of specific IVIG for the treatment of AD may become a reality.

This study had some limitations. There are dozens of phosphorylation sites in tau protein [38]. Instead of exploring the binding of all sites to IVIG, we chose two phosphorylation sites commonly found in AD patients, threonine-181 and Serine-199 [39,40]. Furthermore, the mechanism of IVIG ameliorating LPS-stimulated inflammation is unclear; research to explore this mechanism will be carried out in future studies.

There are notable differences in the concentration of anti-Aβ_42_/tau antibodies and anti-p-tau ratio among these three IVIG products. The inhibitory effects of these IVIGs on systemic and neural inflammation are also different. Combined with our previous study outcomes, the efficacy of IVIG against AD may be positively correlated with its level of AD-related antibodies and anti-inflammatory ability. AD-related antibody analysis and functional evaluation of IVIG should be given sufficient attention before clinical trials, as this may greatly affect the therapeutic effect of AD treatment.

## 4. Materials and Methods

### 4.1. IVIG Selection

Three of the IVIGs that showed significantly different therapeutic effects on 3xTg-AD mice were selected for this study [18]. These IVIGs are produced by manufacturers located in north, northwest, and south China, respectively, and their preparation processes are not identical. These three IVIGs (5%, 50 mL, 2.5 g/bottle) exhibited different neuroprotective effects on 3xTg-AD mice in the previous study [18]: IVIG-A, IVIG-B, and IVIG-C. For 3xTg-AD mice, IVIG-A improved the motor decline in the open-field experiment test and also inhibited the secretion and expression of pro-inflammatory factors; IVIG-B only improved the motor and autonomous decline in the open-field experiment test; and IVIG-C improved cognitive and motor decline in three behavioral outcomes (open-field experiment test, NOR test, Barnes maze test) and inhibited the secretion and expression of pro-inflammatory factors released by the activated glial cell. In brief, from the perspective of the curative effect of IVIG on 3xTg-AD mice, IVIG-C is greater than IVIG-A, and IVIG-A is greater than IVIG-B.

### 4.2. Anti-Aβ_42_ Antibody Assay

#### 4.2.1. Preparation for Aβ_42_ Monomer and Oligomers

As in our previous study [8], Aβ_42_ peptide (AS-24224, AnaSpec, Fremont, CA, USA) was suspended and disaggregated in trifluoroacetic acid (TFA, SHBD1537V, Sigma-Aldrich, Saint Louis, MO, USA) and hexafluoro-2-propanol (HFIP, S2517-10ML, Sigma-Aldrich, Saint Louis, MO, USA). After bath sonication for 1 h, it was resuspended in HPLC-grade water with 0.03% TFA, and Tris base (1346281, Novon, Germany) was added to the concentration of 100 mM. The monomer was obtained by adjusting pH to 8.8 and then centrifuging and filtering. Aβ_42_ peptides were dissolved in HFIP and evaporated naturally after bath sonication for 30 min. The soluble oligomer was obtained by resuspension of dimethyl sulfoxide (D8511 DMSO, Sigma-Aldrich, Saint Louis, MO, USA) for 3~6 days at 4 °C.

#### 4.2.2. Aβ_42_ Conformation Evaluation by Western Blot

Aβ preparations were analyzed via SDS-PAGE using a 4–12% Bis-Tris Gel (22081011, Invitrogen, Camarillo, CA, USA). First, 10 μL of the 4.5 μg/mL monomer and oligomers preparation (0.45 μg) was mixed with 1/3 volume of NuPAGE LDS Sample Buffer (161-0747, BIO-RAD, Hercules, CA, USA) and then loaded into the appropriate lane. After electrophoresis, the proteins were transferred to immobilon PVDF membranes (IPVH00010, Milpore, MA, USA). The membranes were then blocked with Superblock (XJ359126; Thermo Scientific, Rockford, IL, USA) for 2 h at room temperature. Membranes were incubated overnight at 4 °C with agitation in mouse monoclonal anti-Aβ_42_ 6E10 (NBP2-62566; Novus Biologicals, Centennial, CO, USA). After incubation in horseradish peroxidase (HRP)-conjugated anti-mouse IgG (bs-0296G-Bio; Bioss Antibodies, Beijing, China) for 1 h at room temperature with agitation, membranes were developed in SuperSignal West Pico chemiluminescent substrate (WA31601, Thermo Scientifific, Rockford, IL, USA). Bands were visualized with Image Quant LAS 4000 mini.

#### 4.2.3. Anti-Aβ_42_ Monomer and Oligomers Antibodies Concentrations Assay by ELISA 

Concentrations of anti-Aβ_42_ monomer and oligomers antibodies of IVIG were measured by ELISA based on the previous description [8,9]. Aβ_42_ monomer and oligomers were coated with 100 μL/well in 96-well enzyme plate (171376, Nunc Immuno, Thermo Scientific, Roskilde, Denmark) overnight at 4 °C. Diluted IVIG was used as the primary antibody, a mouse anti-human Aβ monoclonal antibody 6E10 (NBP2-62566; Novus Biologicals, Centennial, CO, USA) was diluted as standard (concentration range from 3.9~500 ng/mL), and dilution was used as blank control. Biotinylated goat anti-mouse IgG (bs-0296G-Bio; Bioss Antibodies, Beijing, China) and biotinylated goat anti-human IgG (bs-0296G-Bio; Bioss Antibodies, Beijing, China) were then added separately in wells previously incubated with IVIG preparations and standard samples. The absorbance was read at 450 nm with a microplate reader after adding AP-streptavidin conjugate (1542538A, Invitrogen, Camarillo, CA, USA) and p-Nitrophenyl phosphate (SLCC9851, pNPP, Calbiochem, Merck Millipore, Darmstadt, Germany).

### 4.3. Anti-Tau Antibody Assay

#### 4.3.1. Anti-Tau Antibodies Concentrations Detection by ELISA

Recombinant human tau peptide (tau-441, P4676; Finetest, Hangzhou, China) was brought to room temperature then resuspended in 25 μL TFA followed by 25 μL HFIP. The vial was briefly vortexed. After water bath sonication for 1 h, the contents were dried overnight in a fume hood then stored at −20 °C. The contents of eppendorf (5 μg of tau) were resuspended by vortexing for 3 min in 150 μL HPLC-grade water that had been adjusted to pH 3.0 with TFA (hereinafter referred to as TFA water). Then, 450 μL TFA water was added to the eppendorf with vortexing (estimated concentration of tau = 5 μg tau in 450 μL TFA water = 11 μg/mL). Next, 5.45 mg of Tris base was added to bring the Tris concentration to 100 mM, and 0.95 μL of 12.1 N HCl was then added to adjust to pH 8.8. The contents were centrifuged at 12,000 rpm for 5 min at room temperature. Finally, the supernatant was passed through a 0.22 μm filter (SLGP033RB, Merck Millipore, Boston, MA, USA), put on ice, and used within 1 h, and 11 μg/mL tau were coated with 100 μL/well in a 96-well enzyme plate overnight at 4 °C. Diluted IVIG was used as the primary antibody, and mouse anti-tau monoclonal antibody BT2 (MN1010; Dilution ratio 1:2000; Invitrogen, Camarillo, CA, USA) was diluted as standard (concentration range from 3.9~500 ng/mL), and dilution was used as blank control. Biotinylated goat anti-mouse IgG and biotinylated goat anti-human IgG were then added separately in wells previously incubated with IVIG preparations and standard samples. The absorbance was read at 450 nm with a microplate reader after adding AP-streptavidin conjugate and p-Nitrophenyl phosphate.

#### 4.3.2. Anti-P-Tau Antibodies Ratio Measurement by ELISA

The measurement of anti-p-tau antibody was referred to the previous description with slight modification [41]. A 12 amino acid tau peptide, tau 181–200, which was either non-phosphorylated (“non-p-tau peptide”) or phosphorylated (“p-tau peptide”) at threonine-181 and Serine-199 was purchased from Nanjing YuanPeptide Biotech Ltd., Nanjing, China. Non-p-tau peptide, p-tau peptide, and bovine serum albumin (BSA, Sigma-Aldrich Co., St. Louis, MO, USA) were diluted in Tris buffer (0.1 M, pH 8.8) and were coated with 100 μL/well in 96-well enzyme plate overnight at 4 °C. The diluted IVIG was used as the primary antibody and then added in duplicate to wells previously coated with non-p-tau peptide, p-tau peptide, or BSA. Biotinylated goat anti-human IgG was used as the secondary antibody. The absorbance was read at 450 nm with a microplate reader after adding AP-streptavidin conjugate and pNPP. OD values for wells in which PBS-T-BSA was incubated with non-p-tau peptide, p-tau peptide, or BSA were subtracted from the OD values for wells in which IVIG was incubated with non-p-tau peptide, p-tau peptide, or BSA. Anti-p-tau antibody ratio was calculated as follows:

$$\frac{\mathrm{OD}\;\mathrm{for}\;\mathrm{binding}\;\mathrm{to}\;\mathrm{pTau}\;\mathrm{peptide}-\mathrm{OD}\;\mathrm{for}\;\mathrm{binding}\;\mathrm{to}\;\mathrm{BSA}}{\mathrm{OD}\;\mathrm{for}\;\mathrm{binding}\;\mathrm{to}\;\mathrm{non}-\mathrm{pTau}\;\mathrm{peptide}-\mathrm{OD}\;\mathrm{for}\;\mathrm{binding}\;\mathrm{to}\;\mathrm{BSA}}$$

### 4.4. Anti-Inflammatory Function Evaluation

#### 4.4.1. Animals and Classification

**Figure 5:**
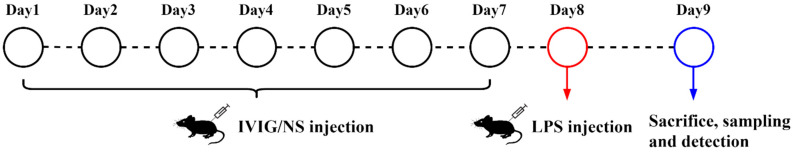
Experimental schedule. NS, normal saline; LPS, lipopolysaccharide.

Seven-week-old Balb/c male mice (*n* = 30) weighing 26 ± 1 g were purchased from a commercial breeder (Dossy Experimental Animals Co., Ltd., Chengdu, China) and used in this experiment. The choice of mouse line refers to the previous description [42,43]. It was found that the inflammatory response induced by LPS in 7-week-old Balb/c mice weighing 26 g was stable and repeatable in the preliminary experiments. Briefly, the mice were maintained under standard conditions (temperature: 22 ± 2 °C, humidity: 40–60%) with a 12 h light/dark cycle and food available ad libitum. After 2 weeks of adjustable feeding, the mice were randomly classified into the following five groups: control (Ctrl) group, LPS (S1732-5 mg, Beyotime, Beijing, China) injection (Model) group, and three IVIG injection (IVIG-A/B/C) groups. IVIG injection group received a prophylactic intraperitoneal injection with 500 mg/kg IVIG and Ctrl/Model group received an equivalent volume of normal saline once daily for 7 days. One hour after the last administration, mice in Model group and IVIG injection groups were intraperitoneally injected with LPS (1 mg/kg), while mice in Ctrl group were injected with the same volume of normal saline (Figure 5).

All experiments complied with the guidelines of Declaration of Helsinki and were approved by the Animal Care and Use Committee of the Institute of Blood Transfusion, Chinese Academy of Medical Sciences (Approval no. 2021048).

#### 4.4.2. Sample Collection and Preparation

Blood, liver, kidney, and brain were collected from the mice after deep anesthesia with 2% sodium pentobarbital. Blood was collected from the inferior vena cava for further assay. The liver, kidney, and brain tissues were removed and then fixed in 4% paraformaldehyde (PFA, BL539A; Biosharp life sciences, Shanghai, China), dehydrated with graded ethanol (100092683, SCRC, Shanghai, China), treated with xylene (10023418, SCRC, Shanghai, China), infiltrated, and embedded in paraffin. Thereafter, coronal sections with a thickness of 5 µm were prepared using frozen microtome (RM2016, Leica Instrument Co., Ltd., Shanghai, China), the sections were mounted on a coated slide, and then the slides were dried on a hot plate at 37 °C overnight. 

#### 4.4.3. WBC, IL-6, and TNF-α Detection in Blood

Blood was collected from the inferior vena cava and divided into two eppendorfs. The contents of one eppendorf were used to detect WBC count by automatic animal five-classification hematology analyzer (XT-1800Iv, Sysmex, Kobe, Japan). The other was centrifuged at 3000 rpm for 20 min to obtain serum, which was quantified for inflammatory factors, such as interleukins (IL)-6 and tumor necrosis factor (TNF)-α by species-specific ELISA kit (VAL604, VAL609; Novus Biologicals, Colorado, USA) using IgG-specific antibodies for capture and the corresponding HRP-conjugated antibodies for detection.

#### 4.4.4. Hematoxylin and Eosin Staining of the Liver and Kidney Tissues

The slides made of the liver and kidney tissues were soaked in Mayer’s hematoxylin (G1003, Servicebio, Wuhan, China) for 30 s, washed with water until clean, and again the slides were soaked in eosin (G1003, Servicebio, Wuhan, China) for 10 s and then washed with water. After air drying, the slides were left at room temperature, then soaked twice each in turn in 95% ethanol, 100% ethanol, 50% ethanol with 50% xylene solution, and then 100% xylene. After air drying at room temperature overnight, the slides were mounted on coverslips using neutral gum (10004160, SCRC, Shanghai, China). Images from the H&E-stained slides were captured using an Image-Pro^®^ Plus computer-assisted imaging system (NIKON DS-U3, Nikon, Shanghai, China) and images were observed with an upright optical microscope (NIKON ECLIPSE E100, Nikon, Shanghai, China). The H-score was used to grade the severity of the lesion of tissues and organs, as shown in Table 1 and Table 2 [32,44]. The liver was evaluated by degeneration, necrosis, inflammatory cell infiltration, and connective tissue hyperplasia, and the kidney was scored by degeneration, inflammatory cell infiltration, necrosis, and bleeding. The scores were calculated by summing all the grades of each item.

#### 4.4.5. Immunohistochemistry of the Brain Tissues

The slides made of the brain tissues were rinsed in PBS and incubated in 3% hydrogen peroxide for 25 min and then blocked for nonspecific antigen binding using 3% BSA for 30 min at room temperature. Subsequently, the sections were incubated with primary antibodies against glial fibrillary acidic protein (GFAP; dilution ratio 1:2000; GB11096; Servicebio, Wuhan, China), and ionized calcium-binding adapter molecule 1 (Iba1; dilution ratio 1:1000; GB113502; Servicebio, Wuhan, China) at 4 °C overnight. On the next day, the sections were washed in PBS and incubated with the appropriate biotinylated secondary antibodies (HRP labeled) from the corresponding species of primary antibody (dilution ratio 1:200; GB23303; Servicebio, Wuhan, China) at room temperature for 50 min. The staining was developed with DAB color developing solution. Finally, the tissues were observed under a microscope and the collagen deposition area was quantitatively analyzed using Image J image analysis software (NIH Image J system, Bethesda, Rockville, MD, USA). The number of positive cells and their staining intensity quantification for each sample was determined by a pathologist blinded to clinical and molecular data using a modified H-score to determine the overall percentage of IBA-1/GFAP positivity across the entire stained brain sample, yielding a range from 0 to 300 [45,46].

H-score: (percentage of weak staining) × 1 + (percentage of moderate staining) × 2 + (percentage of strong staining) × 3.

### 4.5. Statistical Analysis

All data are presented as mean ± SEM and were analyzed with GraphPad Prism 9.0 (GraphPad Software Inc., San Diego, CA, USA) using one-way ANOVA when appropriate, followed by Dunnett’s or Tukey’s post hoc test. * *p* < 0.05, ** *p* < 0.01, ^#^ *p* < 0.001.

## Figures and Tables

**Table 1:** Liver histological scoring parameters.

Grade (Scores)	Condition	Indication
0	Absent	None
1	Very mild	Mild piecemeal necrosis, ballooning degeneration and/or scattered foci of hepatocellular necrosis in lobules or nodules, sprinkling of inflammatory cells in portal tracts (<25%).
2	Mild	Involves 25~50% of the circumference of most portal tracts, involvement of 25~50% of lobules or nodules, increased inflammatory cells in 25~50% of portal tracts.
3	Moderate	Involves more than 50% of the circumference of most portal tracts, involvement of 50% of lobules or nodules, increased inflammatory cells in 50% of portal tracts.
4	Severe	Marked piecemeal necrosis plus bridging necrosis, involvement of >75% of lobules or nodules, dense packing of inflammatory cells in >75% of portal tracts.

**Table 2:** Kindy histological scoring parameters.

Grade (Scores)	0	1	2	3	4
Tubular necrosis	none	0–25%	26–50%	51–75%	76–100%
Tubular dilatation	none	0–25%	26–50%	51–75%	76–100%
Loss of brush border	none	0–25%	26–50%	51–75%	76–100%
Cast formation	none	0–25%	26–50%	51–75%	76–100%

## Data Availability

All data generated or analyzed during this study are included in this published article.
